# Obesity Surgery and Cancer: What Are the Unanswered Questions?

**DOI:** 10.3389/fendo.2020.00213

**Published:** 2020-04-15

**Authors:** Lidia Castagneto-Gissey, James Casella-Mariolo, Giovanni Casella, Geltrude Mingrone

**Affiliations:** ^1^Department of Surgical Sciences, Sapienza University of Rome, Rome, Italy; ^2^Division of Diabetes & Nutritional Sciences, Fondazione Policlinico Universitario A. Gemelli IRCCS, Rome, Italy; ^3^Division of Diabetes & Nutritional Sciences, Università Cattolica del Sacro Cuore Rome, Rome, Italy; ^4^Division of Diabetes & Nutritional Sciences, Faculty of Life Sciences & Medicine, King's College London, London, United Kingdom

**Keywords:** bariatric surgery, cancer incidence, mortality, hormone-sensitive cancer, gastroesophageal cancer

## Abstract

Obesity has become a global epidemic with a soaring economic encumbrance due to its related morbidity and mortality. Amongst obesity-related conditions, cancer is indeed the most redoubtable. Bariatric surgery has been proven to be the most effective treatment for obesity and its associated metabolic and cardiovascular disorders. However, the understanding of whether and how bariatric surgery determines a reduction in cancer risk is limited. Obesity-related malignancies primarily include colorectal and hormone-sensitive (endometrium, breast, prostate) cancers. Additionally, esophago-gastric tumors are growing to be recognized as a new category mainly associated with post-bariatric surgery outcomes. In fact, certain types of surgical procedures have been described to induce the development and subsequent progression of pre-cancerous esophageal and gastric lesions. This emerging category is of great concern and further research is required to possibly prevent such risks. Published data has generated conflicting results. In fact, while overall cancer risk reduction was reported particularly in women, some authors showed no improvement or even increased cancer incidence. Although various studies have reported beneficial effects of surgery on risk of specific cancer development, fundamental insights into the pathogenesis of obesity-related cancer are indispensable to fully elucidate its mechanisms.

## Introduction

The incessant rise of obesity and overweight have configured a state of global epidemic, affecting 1.9 billion and 650 million adults worldwide by 2016, respectively (1). Overall mortality is increased by obesity and its related conditions (2). Amongst these, cancer is indeed the most redoubtable. High body mass index (BMI), namely BMI > 40 kg/m^2^, has clearly been linked to a greater risk of both common a rare malignancy incidence and mortality rates (3, 4).

**Figure 1:**
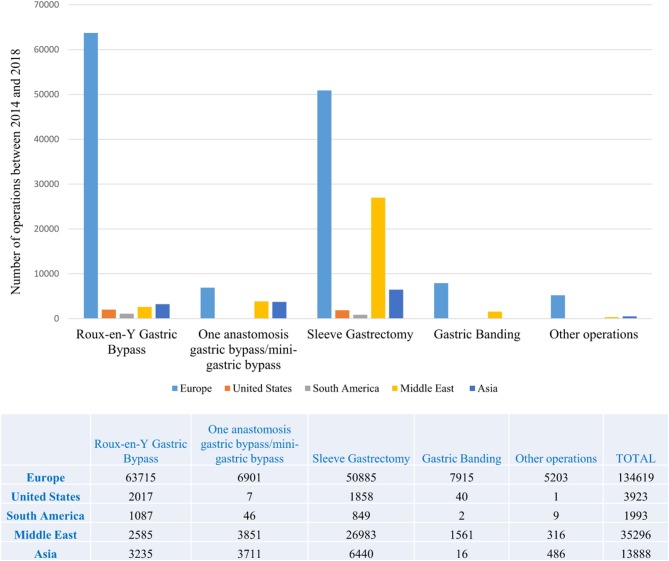
Bariatric/metabolic operations performed worldwide by region between 2014 and 2018 This figure is original and based on data from Welbourn et al. [7].

Obesity-related neoplasms primarily include colorectal and hormone-sensitive (endometrium, postmenopausal breast, prostate) cancers. Bariatric/metabolic surgery (BMS) has been extensively acknowledged to be the most efficacious treatment option for the cure of severe obesity and the number of procedures performed is exponentially growing globally (5–7) (Figure 1). Overall mortality has also been demonstrated to be decreased after BMS (8). On the contrary, it is uncertain whether BMS has any influence over cancer-related mortality.

It is certainly the remission of obesity that has been postulated to be at the basis of cancer incidence reduction or prevention (3, 9–11). Mechanisms involved in obesity-related cancer genesis are multiple and not completely elucidated. Some of these have been suggested to occur through a depauperation of adipose tissue, which causes in parallel a drop in estrogen circulating levels, thereby altering the risk of hormone-sensitive cancers, especially in postmenopausal women (12, 13). Insulin resistance, which is known to often co-exist with obesity, might also have a role in augmented estrogen levels through interaction in insulin signaling pathways, possibly causing exogenous estrogen synthesis (14). Furthermore, several weight loss independent modalities have been proposed to be involved in post-bariatric surgery cancer risk decrease (15) and include lowering of systemic inflammation and inflammatory markers, changes in gastrointestinal hormones, alteration of gastrointestinal anatomy which in turn produces alteration of gut microbiota, fat, glucose and bile metabolism (16).

Available data has generated differing results; while overall cancer risk reduction was reported particularly in women, some authors showed no improvement or even increased cancer incidence.

Herein, we analyze the link between obesity and cancer, reported influence of BMS on outcomes in terms of obesity-associated cancer incidence and mortality and possible mechanisms involved in its genesis.

## Obesity And Overall Cancer Risk

There is a well-established association between the risk of developing several types of cancers and the presence of an increased BMI (i.e., ≥ 25 kg/m^2^) (3). With an ever-growing obesity and overweight prevalence worldwide, it would be sensible to also expect a concomitant rise in cancer incidence.

Approximately 481,000 or 3.6% of all newly diagnosed cancers worldwide in adults aged 30 years or more were considered to be presumably caused by an increased BMI, in 2012. In turn, 13% of all cancers correlated to obesity could be attributable to a raised BMI in the adult population (17, 18). A projected rise of cancer risk development ranging 3-10% was associated to every unit increase in BMI (3). Another projection by the Global Burden of Disease group estimated a 3.9% of all cancer deaths to be linked to excess BMI (19). However, it is likely that these reckonings might be inaccurate due to several possible confounding factors or modifiers such as additional effects of smoking or replacement hormonal therapy. Additionally, most computations do not take into account the latency time, of ~10 years, which elapses between the rise in BMI and the development of a malignancy.

A recent population-based registry study analyzing the incidence of cancer in Nigeria amongst subjects affected by obesity and overweight, found an estimated 1.4%, similarly to other developing countries. Interestingly, when comparing such incidence with other developed countries such as United States, United Kingdom and Australia, this was substantially lower. In fact, the incident cancers attributable to obesity and overweight were 6.0% in the United States, 5.5% in United Kingdom, and 3.4% in Australia, due to a higher prevalence of overweight and obesity in the aforementioned countries (20). Distinctively, North America showed the greatest obesity-related cancer incidence with an ~23% or 111,000 cases, while the Sub-Saharan Africa had the lowest rates of 1.5% or 7,300 cases (17). Hence, it is evident how obesity and overweight rates go hand in hand with cancer risk and that profound variations exist in its incidence according to the countries' level of development.

**Figure 2:**
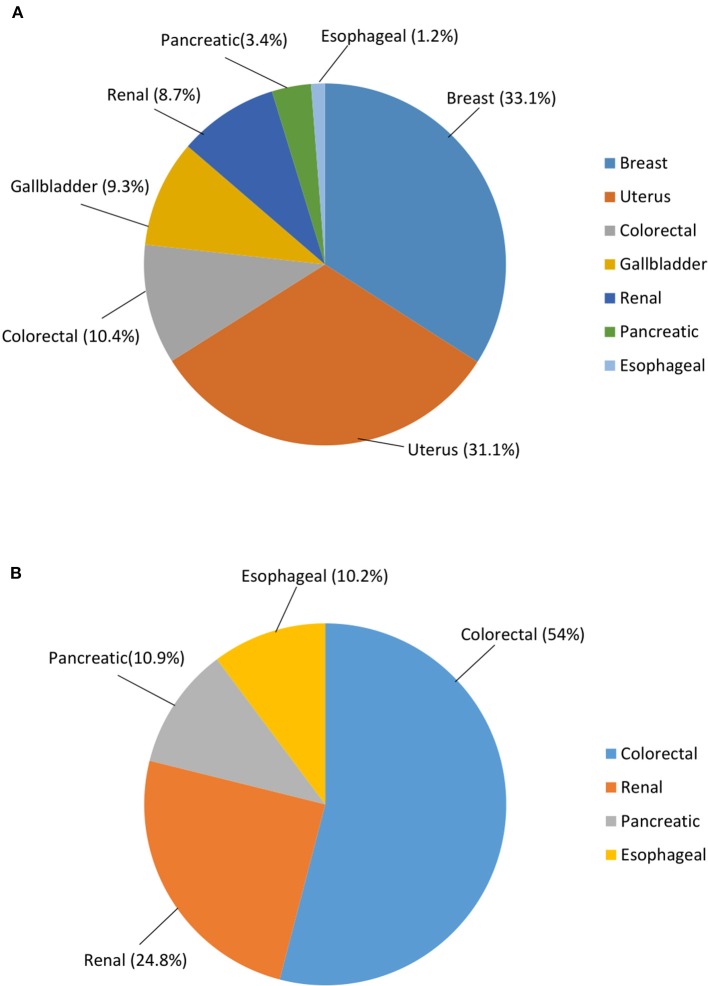
Cancer risk worldwide attributable to overweight and obesity, according to gender. **(A)** Females; **(B)** Males. Data are expressed in %. This figure is original and based on data from Arnold et al. [16].

Excess BMI significantly influences cancer risk according to difference in gender. In fact, while it was estimated to affect 1.9% or 136,000 males in 2012, the risk was more than doubled in females (i.e., 5.4% or 345,000 cases). Furthermore, cancer sites vary greatly amongst genders. Breast (33.1% of all obesity/overweight-related cancers) and uterus (31.1%) were the most represented malignancies in females followed by colorectal (10.4%), gallbladder (9.3%), renal (8.7%), pancreatic (3.4%), and esophageal (1.2%) cancer. On the other hand, the most common obesity/overweight-related cancer in males were colorectal neoplasms (54%) accompanied by renal (24.8%), pancreatic (10.9%), and esophageal (10.2%) cancer (17, 18). Thus, excess BMI-related cancer risk was found to be substantially greater in women compared to men, regardless of the geographical location (Figure 2).

## Obesity-Related Cancer And Systemic Inflammation

Obesity is characterized by a state of low-grade systemic inflammation. This chronic generalized inflammatory condition contributes to the development of metabolic morbidities distinctive of obesity, in addition to possibly mediating cancer genesis (21). Chronic inflammation has been demonstrated to be at the basis of tumor promotion and growth. In fact, inflammatory cells and its mediators have been shown to be present in tumoral tissue and are able to induce cell proliferation and migration, also contributing to neoangiogenesis (22).

Obesity per se is not directly considered to be a cancer risk predictor. In contrast, it is indeed the coexistence of a chronic low-grade systemic inflammation, which is thought to be fundamental in promoting cancer. Confirming this observation, is the fact that “metabolically healthy” obese subjects are characteristically not affected by metabolic dysfunction which is in turn caused by chronic inflammation. In “metabolically unhealthy” obese individuals, instead, an inflammatory status is responsible for the onset of metabolic dysregulation, increased cardiovascular risk and obesity-related cancers (21).

Adipose tissue is growing to be considered as the vastest endocrine organ of the human body, secreting numerous cytokines, adypokines and chemokines. Imbalance between caloric intake and expenditure leads to excessive fat deposition and adipose depot expansion. This adipose depot overgrowth causes tissue dysfunction and alteration of its histology due to increased apoptosis, macrophage recruitment and release of several pro-inflammatory molecules, typical of adipose inflammation. Consequently, this contributes to peripheral insulin resistance, hyperglycemia, dyslipidemia, all of which are involved in inducing oxidative stress, cancer development and sustainment (23).

It is important to note how not all types of inflammatory processes are linked to cancer. In actual fact, a distinctive feature of acute inflammation is the presence of natural killer (NK) and CD8+ T cells at the interested site, also involved in tumor immunity. On the contrary, chronic inflammatory sites are lacking these types of cell populations (24). This tissue is instead characterized by a reduction of anti-inflammatory T_H_2 and regulatory T cells (Treg) with an increase in the population of T_H_1 and CD8+ T cells, in addition to a shift of the M2:M1 macrophage ratio (25). It is this very same type of chronic inflammation of the adipose tissue, which is involved in metabolic dysfunction, cardiovascular risk and carcinogenesis.

**Figure 3:**
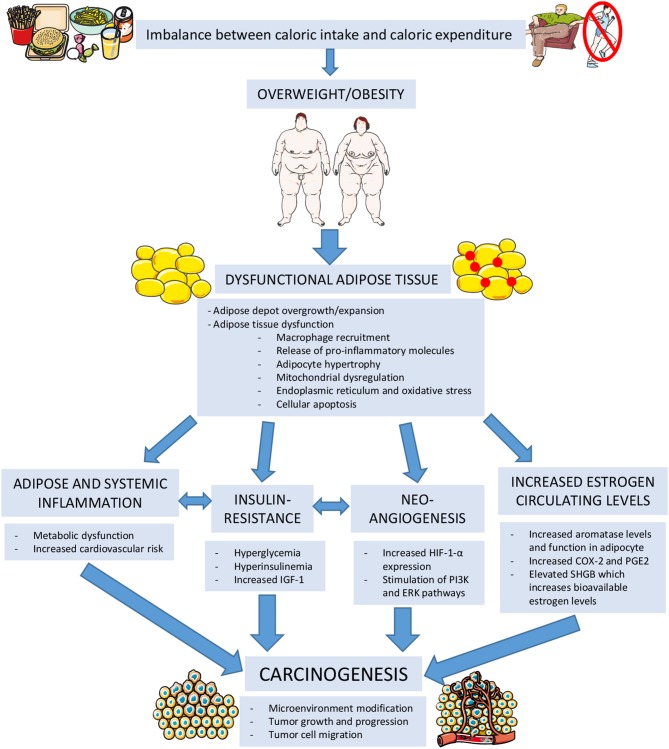
Mechanisms linking obesity to carcinogenesis. IGF-1, Insulin-Like Growth Factor 1; HIF-1-α, Hypoxia-Inducible Factor 1 Alpha; PI3K, Phosphoinositide 3-kinase; ERK, extracellular signal-regulated kinase; COX-2, Cycloxygenase 2; PGE2, Prostaglandin E2; SHBG, Sex-Hormone Binding Globulin. (The figure was created using Servier Medical Art).

A balance between anti-inflammatory and pro-inflammatory factors and immune system cells is present in healthy lean adipose tissue and is capable of maintaining its normal storage capacity, endocrine and whole-body metabolic function. Alteration of this fine equilibrium is responsible of adipocyte hypertrophy, mitochondrial dysregulation, endoplasmic reticulum and oxidative stress, ultimately leading to the release of pro-inflammatory factors and finally cellular apoptosis. This configures a state of adipose inflammation. Adipose tissue ability to store energy and to perform its normal endocrine functions is deeply hampered in obese subjects. In regular conditions, stromal cells are responsible of suppressing tumor growth. Adipose inflammation and the altered adipokine and cytokine secretion can modify this microenvironment and also promote tumor growth, progression and tumor cell migration. Additionally, this inflammatory state causes insulin resistance which translates into a state of hyperglycemia, hyperinsulinemia, increased circulating insulin-like growth factor 1 (IGF-1) which have also been recognized to be involved in carcinogenesis (25) (Figure 3).

Local inflammation seems to also play a substantial role especially in breast cancer development. White adipose tissue inflammation in the breast causes histological alterations and release of NFκB, which leads to augmented aromatase activity and estrogen-androgen ratio in the breast tissue. Local and systemic raised estrogen levels are ideal for tumor formation and progression (21).

Epigenetic variations in DNA over time have also been proposed among chief determinants linking obesity and cancer. Several environmental factors, including nutrition, physical exercise and lifestyle, may be involved in such mechanisms, via the activation of inflammatory processes (26). Recent investigations in animal models have shown how diet-induced obesity can decrease DNA methylation and downregulate expression of certain genes, promoting breast cancer with a worsened prognosis. Preventive measures and strategies targeting epigenetic changes are starting to be developed in order to disrupt this linkage (27).

## Obesity-Related Cancer And Insulin Resistance

Central obesity measured by waist circumference has been found to be a direct predictor of cancer risk. In other words, it is visceral fat rather than peripheral adipose tissue which is correlated with this very risk (28, 29). To this regard, visceral adipose tissue (VAT) has increased lipolytic and lipogenic action additionally to its greater pro-inflammatory factors secretion, compared to subcutaneous adipose tissue (SAT). Metabolic dysfunction and cardiovascular risk are both associated with greater VAT depots (29).

Excess body weight contributes to inducing a state of insulin resistance, characterized by augmented hepatic gluconeogenesis, reduced peripheral glucose uptake and hyperinsulinemia. Prolonged hyperinsulinemia may lead to increased IGF-1 secretion (30). *In vitro* studies have demonstrated the fundamental role of IGF-1 and insulin itself in stimulating tumoral cell growth. Similarly, epidemiological investigations have shown a direct correlation between increased insulin and IGF-1 circulating levels and the development of certain types of malignancies (i.e., pancreatic, liver, colorectal, breast, and endometrium). Furthermore, IGF-1 is capable of promoting tumoral development and sustainment, also stimulating hypoxia-inducible factor 1 alpha (HIF-1-α) release that is associated with specific tumor migration and metastasis and may inhibit certain tumor suppressor genes such as p53. Insulin, on the other hand, also has a role in tumor progression and metastasis through stimulation of different intracellular pathways comprising PI3K and ERK (31, 32) (Figure 3).

Mounting evidence has additionally shown that cancer prognosis is affected by insulin and IGF-1 circulating levels, independently of cancer risk. Specifically, a state of hyperinsulinemia is associated with worst cancer prognosis and outcomes. In consideration of the ever-growing obesity global epidemic, insulin resistance and hyperinsulinemia, closely interrelated with obesity, are receiving increasing attention and new experimental antineoplastic drugs—targeting IGF-1 and/or insulin signaling pathways—are being employed in clinical trials (31).

## Obesity-Related Cancer And Circulating Estrogen Levels

There is a rise in estrone, free and bound estradiol levels in parallel to a rise in BMI (33). The main source of aromatase in the human body resides in preadipocytes, adipocytes, and mesenchymal stem cells found in the adipose tissue. This enzyme is responsible for transforming androgens to estrogens. Levels and function of aromatase increase proportionally to adiposity and aging, with adipose tissue becoming the main secretion site in postmenopausal women. Furthermore, chronic adipose inflammation produces an increased cycloxygenase-2 (COX-2) expression and prostaglandin E_2_ (PGE_2_) secretion which combines with the already high levels of local pro-inflammatory cytokines; this scenario is capable of inducing aromatase expression and augmented estrogen production, configuring an optimal microenvironment for carcinogenesis. Eicosanoids have also shown to generate tumor cell migration, neoangiogenisis and cell growth, due to its anti-apoptotic and pro-mitotic pathways (34).

Sex-hormone binding globulin (SHBG) levels are also profoundly affected by the amount of adipose tissue as they decrease when adiposity rises. A reduction in SHBG concentration causes a greater portion of estrogen to be bioactive and readily available (35).

Hence, obesity is responsible of increasing estradiol production in adipose tissue and elevating the fraction of biologically active estrogen by influencing SHBG synthesis by the liver (34). The augmented concentration of total serum estrogens and low SHGB levels have been demonstrated to have a causal relationship with postmenopausal breast and endometrial cancer risk development (Figure 3).

## Obesity-Related Cancer And Gut Microbiota

Over the past decade, a large body of literature has been dedicated to investigating the role of gut microbiota in the physiopathological mechanisms involved in metabolic regulation in the ambit of cardio-metabolic disorders. Gut microbiota is mostly present in the ileum and colon and is involved in the host defensive action from pathogens, growth and development of the digestive tract, immune response, balance of energy homeostasis, and digestion of nutrients. Its composition is greatly influenced by genetic background, diet, exercise and antibiotics. Obesity and type 2 diabetes mellitus in humans have been linked to alteration in gut microbiota composition. In turn, through energy harvesting of dietary nutrients, gut microbiota can indirectly affect insulin signaling and systemic low-grade inflammation (36). A direct relationship between gut microbiota and gastrointestinal cancers has been shown to occur by way of systemic inflammation and immune response regulation. The gut barrier dysfunction was proven to activate several different pathways, able to promote the development of certain gastrointestinal cancers, including colorectal, hepatocellular and pancreatic cancers (37). BMS induces changes in gut microbiota, which is involved in weight loss, fat deposition normalization, indirectly contributing to cancer risk reduction (36).

## Obesity-Related Cancer And Non-Alcoholic Fatty Liver Disease

Non-alcoholic fatty liver disease (NAFLD) includes a clinical continuum of hepatopathies that range from simple steatosis to non-alcoholic steatohepatitis (NASH) and possibly evolving to liver fibrosis, cirrhosis and hepatocellular carcinoma (HCC). As a result of the ever-rising incidence of overweight, obesity and type 2 diabetes mellitus worldwide, NAFLD has become the most commonly diagnosed liver condition in industrialized countries. Approximately 25% of subjects with NASH develop progressive fibrosis and subsequent cirrhosis (38). Several authors have demonstrated how NAFLD has presently become the main risk factor for the development of HCC, compared to previously recognized predisposing causes such as hepatitis B and C infection (39).

BMS is recommended in those patients affected by morbid obesity in which pharmacotherapy and lifestyle modifications have failed. However, no randomized controlled trials (RCTs) comparing BMS with standard of care have been published until now. A prospective study including 109 subjects demonstrated histological resolution of NASH 1 year after RYGB, bilio-intestinal bypass or gastric banding in 85% of the cases (40). The same authors (41) showed in 381 subjects undergoing liver biopsy during BMS, mainly RYGB, that the percentage of subjects with NASH declined from 27.4 to 14.2% at 5 years, even though, fibrosis somewhat worsened.

A recent meta-analysis (42) including 3093 liver biopsies demonstrated resolution of steatosis in 66%, inflammation in 50%, ballooning in 76%, and fibrosis in 40% of the cases. However, in 12% of subjects fibrosis worsened if present at the baseline or appeared if absent.

The reduction of NAFLD or NASH prevalence in subjects operated of BMS should translate in a future reduction in the incidence of HCC.

## Effects of Bariatric/Metabolic Surgery on Obesity-Related Cancer

Bariatric/metabolic surgery seems to have protective effects with regards to cancer development and prognosis. However, this anticarcinogenic action has been shown to be multifactorial and may include weight-dependent and independent mechanisms. Interestingly, several studies have shown a lack of correlation between the entity of weight loss after BMS and the decrease in cancer incidence (15, 43). This is to further confirm how BMS exercises its effects not solely through excess weight loss, but is rather likely to be mediated by various mechanisms. These might include substantial reduction in pro-inflammatory molecule secretion, alteration of gut microbiota, changes in glucose and fat metabolism, improvement of insulin sensitivity, modification of gastrointestinal peptides (15, 21, 24, 31, 32).

Some authors found beneficial effects of BMS in terms of reduction of cancer incidence and mortality in females, especially in the postmenopausal subgroup; the same benefit was not proven in males (43, 44). Such positive outcomes in females are likely to be associated with a substantial decrease in estrogen production and bioavailability, translating in a decline of hormone-sensitive breast and endometrial cancers after BMS (15).

A 2-cohort observational study by Christou et al. comparing patients who received BMS to a control group who did not undergo surgery, demonstrated in parallel with an excess weight loss of 67.1%, a 76% overall reduction of hospital visits for all types of cancers in the post-bariatric group (2.0 and 8.45%, respectively). Moreover, relative risk of breast cancer incidence in the study group had an additional 82% reduction (45). Similarly, Adams et al. conducted a retrospective analysis assessing the effects of BMS (specifically, gastric bypass) compared to non-surgical obese patients, on cancer incidence and mortality over a 24-year period. Significantly lower cancer frequency was found in the surgical group compared to non-operated controls, with cancer-specific mortality decreased by 46% in the former group. Although cancer incidence declined only for obesity-related malignancies (i.e., esophagus, colorectal, pancreas, postmenopausal breast, uterus, kidney, liver, gallbladder), mortality dropped for all cancer types (45, 46).

McCawley et al. further investigated the influence of BMS in female obese subjects in a retrospective study compared to a non-surgical control group. Both groups were found to be younger in age at diagnosis in comparison to the general non-obese population. Furthermore, the non-operated control group had a higher incidence of cancer rates compared to operated subjects (5.8 vs. 3.6%, respectively). Most commonly diagnosed malignancies in both groups were breast, endometrial, cervix and colorectal cancers (47).

The Swedish Obese Subjects (SOS) study was the first prospective controlled intervention trial to evaluate the impact of BMS on cancer rates and outcomes after a mean 10.9-year follow up period. Authors concluded that surgical patients had substantially lower cancer prevalence, however, this was confirmed in women but not the same was found in operated men (43).

A systematic review and meta-analysis involving six observational studies comparing outcomes in terms of cancer risk and mortality in surgical vs. non-surgical obese subjects concluded that BMS is capable of reducing overall cancer incidence and related mortality. Nevertheless, after stratifying patients by gender, beneficial effects of surgery were seen in women but not confirmed in post-bariatric surgical men (48).

To further investigate the observation that substantial weight loss might prevent the risk of developing endometrial hyperplasia and type 1 endometrial cancer, Argenta et al. performed a prospective, blinded, non-interventional pilot study on 45 premenopausal morbidly obese women candidate to BMS. Authors found endometrial pathology (simple or complex endometrial hyperplasia) in 6.8% of the study group at baseline and observed a complete regression of the endometrial lesions in all patients 12–18 months postoperatively. In this group of patients a significant risk reduction of endometrial malignancies was recorded but not abolished (49). Clinical trials are currently underway to establish whether bariatric surgery coupled with hormonal therapy is an effective treatment for endometrial cancer/atypical hyperplasia in obese women of childbearing age who wish to maintain their fertility and might help to unravel possible molecular mechanisms causing endometrial cancer reduction after BMS.

A Swedish nationwide population-based cohort study on 13,123 subjects, by Ostlund et al. analyzed the effect of BMS on long-term obesity-related cancer (breast, prostate, colorectal, endometrial, and kidney) risk and mortality over a 26-year period, calculated by a standardized incidence ratio (SIR). In contrast to the aforementioned studies, Ostlund et al. failed to show a protective effect of surgery on oncologic outcomes in the long term after BMS. Specifically, authors found no reduction of SIRs after BMS over time for all obesity-related cancers, except for colorectal cancer, which had an increased SIR in the long-term for post-bariatric subjects (50).

Colorectal cancer has been shown to be closely associated with obesity, increasing alongside the rise in BMI. It has been suggested that for every increase of 5 kg/m^2^ in BMI, colorectal cancer risk increases by 24% in males and 9% in females (3). A recent systematic review and meta-analysis by Afshar et al., analyzing the effects of BMS solely on colorectal cancer incidence, demonstrated a significantly diminished risk, by an estimated 27% in obese operated patients compared to controls (49, 51). However, data in literature regarding this specific risk is inconsistent. Several authors have highlighted the protective effects of BMS on overall cancer risk (3, 36, 45). However, the majority of studies did not demonstrate adequate statistical power nor sufficient length of follow up, which is needed especially for colorectal malignancies, which do have indeed a long decennary carcinogenetic process.

Similarly to findings by Ostlund et al. (50), Derogar et al. (52) highlighted the rise in SIRs for colorectal cancer in the post-bariatric surgical group composed of 15,095 patients, which increased concurrently with time after surgery, compared to the non-operated group (62,016 subjects) who's SIRs continued to be stable also in the long term. Interestingly, authors did not find any difference in SIRs for colorectal cancer in-between the different surgical procedures analyzed in the study (i.e., VBG, AGB, and RYGB). The grounds for such increase is unclear, but the same authors also demonstrated in a previous study how patients who underwent RYGB developed rectal mucosal hyperproliferation persisting at least 3 years postoperatively. Furthermore, a raised mucosal expression of the pro-tumorigenic cytokine macrophage migration inhibitory factor was found (53). It has been hypothesized that colorectal cancer development might increase as a consequence of the malabsorptive effects caused by certain bariatric procedures, where RYGB is the most studied one. The altered intestinal absorption and the surgical bowel rearrangement might lead to the increased mucosal bile salt exposure and modification of gut microbiota. Additionally, the modified dietary intake after surgery, principally composed of proteins and low in carbohydrates, can contribute to the production of harmful metabolites in the colonic tract with possible cytotoxic effects on the mucosa and playing a role in the complex interaction with gut microbiota and bile salts (54, 55).

Existing evidence derives largely from observational retrospective studies, which might represent a selection bias and may influence the reliability of results. Nevertheless, the vast majority of studies available in literature undeniably highlight the positive impact of BMS on specific cancer incidence, prognosis and mortality. This may occur through several pathways that are correlated to weight loss but also involve multiple mechanisms that go beyond mere weight reduction and that seem to be also associated to metabolic modifications that occur after surgery.

## Bariatric/Metabolic Surgery and New onset of Gastroesophageal Cancer

Overweight and obesity have been linked to a raised risk of esophageal and gastric cancer development and this has been shown to increase along with rising BMI (56). This correlation (particularly present for malignancies of the distal esophagus and cardia) has been attributed to the higher coexistence in this population of gastroesophageal reflux disease (GERD), hiatal hernia and subsequent erosive esophagitis of variable degrees which may seldom evolve to Barrett's esophagus (57, 58). Helicobacter pylori infection concurrently plays a role in cancer development, especially for gastric malignancies and is in fact classified as a type 1 carcinogen (59). Additionally, hormonal effects generated by dysregulation of insulin and IGF-1 secretion, generally altered in obese subjects, may be implicated in this carcinogenic process (30, 31).

By surgically inducing weight loss, BMS could presumably reduce some risk factors implicated in gastroesophageal tumorigenesis. However, scarce and contrasting evidence has been reported in literature to this regard.

Bariatric procedures encompassing a restriction of the gastric outlet, such as vertical banded gastroplasty (VBG), adjustable gastric banding (AGB), and sleeve gastrectomy (SG), are characterized by certain pathophysiologic modifications possibly involved in gastroesophageal malignancy development. These might comprise alimentary bolus stasis and impaction in the esophagus or gastric pouch due to the presence of a narrowed outlet, local inflammation adjacent to the gastric band location, alteration of gastroesophageal intraluminal pressures and motility with consequent appearance of differing types of gastroesophageal reflux.

The protracted stasis of food content in the distal esophagus or gastric reservoir might expose the mucosa to the harmful effects of exogenous carcinogens (60, 61). Furthermore, certain studies have investigated the local alterations arising in correspondence to the gastric band or mesh (i.e., VBG, ABG). The presence of a foreign body in fact, causes the formation of adhesions, scar retraction, erosion, ulceration, local blood flow reduction and mucosal changes leading to metaplasia that in rare cases may evolve to dysplasia and adenocarcinoma (62–64).

The role of chronic GERD has been proven to have a fundamental role in the genesis of gastroesophageal malignancies. Due to this, extensive research has been dedicated to further understand the effects of BMS on GERD. Specifically, SG was the bariatric procedure which raised the greatest concerns in consideration of its exponentially rising popularity. The effects of SG on GERD are rather conflicting (65). However, several studies have demonstrated that SG substantially causes de novo or a worsening of preexisting GERD, development of erosive esophagitis and hiatal hernia as evaluated by the use of endoscopy (66, 67). Interestingly, no correlation was found between the presence of GERD symptoms and endoscopic findings; indeed authors demonstrated how the vast majority of patients often were asymptomatic for pyrosis, reflux or regurgitation (68). Furthermore, a peculiarity of SG is that the composition of refluxate undergoes profound modifications after surgery and is characterized by the greater presence of bile (66, 67). Our previously published data on endoscopy before and after SG actually confirmed the consensual increase of bile stagnation into the esophagus, which strongly correlated with the degree of erosive esophagitis and Barrett's metaplasia (66). Earlier investigations suggest that biliary or mixed types of gastroesophageal reflux are responsible for esophageal mucosal injury contributing to the development of Barrett's esophagus (69). Several studies actually reported a significantly elevated number of Barrett's esophagus after SG (66, 67, 70), reaching a prevalence as high as 18.8% 5 years after this bariatric operation (70). Barrett's esophagus is considered to be a precancerous lesion and some authors have suggested that this histological alteration might evolve to dysplasia even more rapidly after SG (66, 69). Esophageal adenocarcinoma is in fact the most common malignancy emerging after SG and this might occur on previous metaplasia. However, to this regard, reported cases in literature are sporadic and do not allow to make definitive conclusions on the actual prevalence of such malignancies (70).

After bypass surgery, namely, Roux-en-Y gastric bypass (RYGB), the second most commonly performed bariatric operation, several authors have described tumors arising mainly from the gastric remnant. The excluded stomach might be a site of carcinogenesis due to the accumulation of pancreatic and biliary content at this level that has been shown to generate in experimental studies the progression to intestinal metaplasia and adenocarcinoma (71). Helicobacter Pylori infection has been clearly linked to gastric cancer and some studies have shown how this infection may persist in the gastric remnant after RYGB despite eradication (72). This further supports the importance of its treatment and eradication prior to RYGB (73).

One anastomosis gastric bypass (OAGB) has gained popularity over the past five years and was recently accepted as a standard bariatric procedure by the International Federation for the Surgery of Obesity and Metabolic Disorders (IFSO). This procedure has been hypothesized to potentially cause the development of gastric cancer, owing to its peculiar bypass reconstruction. Previous studies have clearly shown the appearance of gastric adenocarcinoma at the level of the gastro-jejunostomy after Billroth II reconstruction (74, 75). Considering the fact that in OAGB a similar loop reconstruction is performed, it could presumably be linked to a similar carcinogenetic risk to that seen after Billroth II (76). Furthermore, biliary reflux in the distal esophagus is a major concern as this has been shown to be associated with increased incidence of metaplasia, dysplasia, and finally esophageal adenocarcinoma (69). Nevertheless, data in literature is widely lacking due to the relatively recent nature of this procedure.

Preoperative endoscopic screening is of paramount importance in order to rule out possible intraluminal pathological alterations, including erosive esophagitis, hiatal hernia, Barrett's esophagus, malignant tumors, etc. This evaluation is also necessary to properly assess patients who are often asymptomatic for GERD, might have the aforementioned conditions and might go unnoticed if a preoperative endoscopy is not performed. A proper and complete examination, in fact, can contribute to the correct patient selection and submission to the best bariatric operation in that specific case, also contributing to the reduction of postoperative complications.

The reported cases of esophagogastric cancers after BMS are sporadic and insufficient, not allowing to reliably draw a relationship between such types of malignancies and each bariatric surgical procedure. Additionally, epidemiological studies do not show a greater incidence of esophageal and gastric cancers in post-bariatric subjects compared to the general population (77). Nevertheless, higher-level evidence is necessary to fully understand and eventually confirm or deny any intercorrelation. Endoscopic surveillance after all types of bariatric operations seems to be of paramount importance for early detection of esophagogastric malignancies.

## Obesity Surgery and Cancer: What are the Unanswered Questions?

### Which Types of Cancers Benefit More From BMS?

BMS appears to have protective effects on cancer development. This anticarcinogenic action is multifactorial and includes weight-dependent and independent mechanisms. There is a lack of correlation between the entity of weight loss after BMS and the decrease in cancer incidence. This is to confirm how BMS exercises its effects not solely through weight loss, but is rather likely to be mediated by various mechanisms (15).

The beneficial effects of BMS in terms of reduction of cancer incidence and mortality has especially been shown in females, specifically in the postmenopausal group (12, 13). Such positive outcomes in females are likely to be associated with a substantial decrease in estrogen production and bioavailability, translating in a decline of hormone-sensitive breast and endometrial cancers after BMS (34).

### Why Does BMS Benefit More Women Than Men?

It is certainly the remission of obesity that has been postulated to be at the basis of cancer incidence reduction or prevention. Mechanisms involved in obesity-related cancer genesis are multiple and not completely elucidated. Some of these have been suggested to occur through a depauperation of adipose tissue, which causes in parallel a drop in estrogen circulating levels, thereby altering the risk of hormone-sensitive cancers, especially in postmenopausal women (12–15). Insulin resistance, which is known to often co-exist with obesity, might also have a role in augmented estrogen levels through interaction in insulin signaling pathways, possibly causing exogenous estrogen synthesis. Furthermore, several weight loss independent modalities have been proposed to be involved in post-bariatric surgery cancer risk decrease and include lowering of systemic inflammation, changes in gastrointestinal hormones, alteration of gastrointestinal anatomy which in turn produces alteration of gut microbiota, fat, glucose, and bile metabolism. Hence, hormonal levels are at the basis of the improved cancer risk especially in postmenopausal women (34, 35). However, several mechanisms involved in this phenomenon are still waiting to be clarified.

### Does Only Cancer Risk Improve or Is Also Prognosis Affected After BMS?

BMS not only affects cancer incidence but has also been shown to profoundly influence cancer prognosis. This may occur through several pathways that are in part correlated to weight loss but also involve multiple mechanisms that go beyond mere weight reduction and that seem to be associated to metabolic modifications occurring after surgery. In fact, evidence suggests that cancer prognosis is affected by insulin and IGF-1 circulating levels, independently of cancer risk. Specifically, a state of hyperinsulinemia is associated with worst cancer prognosis and outcomes. In consideration of the ever-growing prevalence of obesity and insulin resistance, an increasing interest to the development of new experimental antineoplastic drugs—targeting IGF-1 and/or insulin signaling pathways—are being employed in clinical trials (14, 32).

### Which Operation Should Be Recommended for Each Specific Type of Cancer?

No evidence is available at present regarding the beneficial effects of different bariatric procedures on specific cancer types. In consideration of this, the various surgical bariatric procedures available should be advised by the bariatric surgeon based on the preoperative work-up and available institutional and international guidelines (78).

### Does GERD Contraindicate Specific Bariatric Procedures?

Currently, GERD, regardless of its severity, is not considered as an absolute contraindication to any specific bariatric procedure. However, several bariatric surgeons may oftentimes advice the patient to undergo operations such as RYGB, which is most effective in leading to GERD resolution, as opposed to “refluxigenic” procedures such as SG (78, 79). In fact, SG is controversially associated with an increase or worsening of GERD and the appearance of hiatal hernia and erosive esophagitis. The role of chronic GERD has been proven to have a fundamental role in the genesis of gastroesophageal malignancies. It is for this very reason that SG is often not recommended in patients who have pre-existing GERD. Several studies actually reported a significantly elevated number of Barrett's esophagus after SG. Barrett's esophagus is indeed a precancerous lesion and some authors have suggested that this histological alteration might evolve to dysplasia even more rapidly after this operation (66, 67). Esophageal adenocarcinoma is in actual fact the most common malignancy emerging after SG and postoperative endoscopic surveillance is fundamental for early diagnosis in this group of patients (69, 70, 77).

## Conclusions

Available data has shown that obesity is indeed associated with an increased cancer risk and BMS is capable, through several diverse pathways, to generate a significant reduction in overall cancer prevalence and mortality. However, awareness should be raised with regards to the possibility of increasing the incidence of gastroesophageal cancers after BMS and the necessity of postoperative endoscopic surveillance. Further understanding of involved mechanisms in the development or reduction of such neoplasms in this specific type of patients is required in order to possibly formulate public health strategies in such setting.

## Author Contributions

All authors listed have made a substantial, direct and intellectual contribution to the work, and approved it for publication.

### Conflict of Interest

The authors declare that the research was conducted in the absence of any commercial or financial relationships that could be construed as a potential conflict of interest.

## Abbreviations

BMI: Body Mass Index
BMS: Bariatric/Metabolic Surgery
NK: Natural Killer
Treg: Regulatory T Cells
IGF-1: Insulin-Like Growth Factor 1
VAT: Visceral Adipose Tissue
SAT: Subcutaneous Adipose Tissue
HIF-1-α: Hypoxia-Inducible Factor 1 Alpha
COX-2: Cycloxygenase 2
PGE2: Prostaglandin E2
SHBG: Sex-Hormone Binding Globulin
NAFLD: Non-Alcoholic Fatty Liver Disease
NASH: Non-Alcoholic Steatohepatitis
HCC: Hepatocellular Carcinoma
SOS: Swedish Obese Subjects
SIR: Standardized Incidence Ratio
GERD: Gastroesophageal Reflux Disease
VBG: Vertical Banded Gastroplasty
AGB: Adjustable Gastric Banding
SG: Sleeve Gastrectomy
RYGB: Roux-en-Y Gastric Bypass
OAGB: One Anastomosis Gastric Bypass
IFSO: International Federation for the Surgery of Obesity and Metabolic Disorders.
